# *Guazia*, the earliest ovule without cupule but with unique integumentary lobes

**DOI:** 10.1093/nsr/nwab196

**Published:** 2021-10-30

**Authors:** De-Ming Wang, Le Liu, Yi Zhou, Min Qin, Mei-Cen Meng, Yun Guo, Jin-Zhuang Xue

**Affiliations:** Key Laboratory of Orogenic Belts and Crustal Evolution, Department of Geology, Peking University, Beijing 100871, China; School of Geoscience and Surveying Engineering, China University of Mining and Technology (Beijing), Beijing 100083, China; Key Laboratory of Orogenic Belts and Crustal Evolution, Department of Geology, Peking University, Beijing 100871, China; Institute of Geology and Paleontology, Linyi University, Linyi 276000, China; Key Laboratory of Orogenic Belts and Crustal Evolution, Department of Geology, Peking University, Beijing 100871, China; Institute of Deep Time Terrestrial Ecology, Institute of Palaeontology, Yunnan Key Laboratory of Earth System Science, Yunnan University, Kunming 650500, China; Key Laboratory of Orogenic Belts and Crustal Evolution, Department of Geology, Peking University, Beijing 100871, China

**Keywords:** ovule, cupule, integument, Devonian, seed plants

## Abstract

The earliest ovules in the Late Devonian (Famennian) are surrounded by a cupule that is involved in both protection and pollination, and generally have free integumentary lobes. Here we report a new taxon from the Famennian of China, *Guazia dongzhiensis* gen. et sp. nov. The terminally borne ovule is apparently acupulate (without cupule) and has four radially arranged wing-like integumentary lobes that are extensively fused, and folded lengthwise and inwards. *Guazia* provides evidence that not all Devonian seeds possess a cupule and it increases their diversity in integumentary lobes. This genus also suggests that the integuments develop new functions, probably including wind dispersal at the expense of the cupules.

## Teaser

The Late Devonian ovule or seed reported in this paper lacks cupule, which was believed to characterize the earliest seed plants in the Famennian. Instead, the unique integument functions as protection, pollination and probable wind dispersal.

## INTRODUCTION

The earliest ovules or seeds (fertilized ovules) in the Late Devonian (Famennian, 372–359 million years ago [Ma]) have been reported from Europe, North America and China [1]. The cupule, derived from fusion of a vegetative branching system, is an accessory structure with free or united segments surrounding one to several ovules [2,3]. Famennian ovules are (usually) borne within a segmented cupule [4] that is thought to have functioned as protection while also aiding in pollen capture [2,5].

Famennian ovules are characterized by an integument (developing into a seed coat covering the megasporangium termed nucellus) that is variously lobed, more or less free from the nucellus and serves to increase protection and pollination [2]. These ovules lack a well-defined micropyle as a small hole in the more complete integument, which was evolved by later seed plants (spermatophytes) and through which the pollen enters [6].

In this study, a new taxon, *Guazia dongzhiensis* gen. et sp. nov., from China is known to have borne terminal ovules on the branches and represents the oldest acupulate ovule. Hitherto, *Warsteinia* from Germany was the only Famennian ovule with four wing-like integumentary lobes that may have been adapted for wind dispersal (anemochory), but its attachment and cupule remain unclear [7,8]. *Guazia* is shown to be another Famennian ovule with four radially symmetrical wing-like integumentary lobes. Its acupulate ovule with unusual integument provides new insights into the early evolution of seed plants.

## RESULTS

### Locality and stratigraphy

The plant fossils were collected from the Wutong (Wutung) Formation at Xiangkou Section (30°03^′^57^′′^N and 116°47^′^26^′′^E), Xiangyu Town, Dongzhi County, Anhui Province, China (Fig. S1A–C). This formation, widespread in the lower reaches of the Yangtze River including Anhui, consists of the Guanshan Member characterized by quartzose sandstone and conglomerate, and the overlying Leigutai Member with inter-beds of quartzose sandstone and mudstone [9]. Assemblages of plants, fish, conchostracans and especially spores indicate that the Wutong Formation (possibly excluding the uppermost part) is Upper Devonian (Famennian) [9–11]. At the Xiangkou Section, the progymnosperm *Archaeopteris halliana* occurs in the basal part of the Leigutai Member [12]. *Guazia dongzhiensis* and a pollen organ *Placotheca minuta* [13] were found separately ca. 10 m and 11 m above the *A. halliana* horizon (Fig. S1D). Another pollen organ *Telangiopsis* sp. [14] sometimes occurs together with *G. dongzhiensis*. *Archaeopteris* was distributed worldwide in the Late Devonian [2,15]. These data indicate a Famennian age for *G. dongzhiensis*. From a layer of muddy siltstone, we obtained ∼300 specimens of ovules preserved as impressions or compressions with coaly material preserved.

### Systematic paleontology

Division: Spermatophyta

Order and family: incertae sedis

*Guazia dongzhiensis* Wang, Liu, Zhou, Qin, Meng, Guo and Xue gen. et sp. nov.

Etymology: the generic name is from ‘Guazi’, the Chinese pinyin for sunflower seeds with a shell, referring to the shape of the organ; the specific epithet is derived from Dongzhi, the name of the county where the fossils were collected.

**Figure 1.**
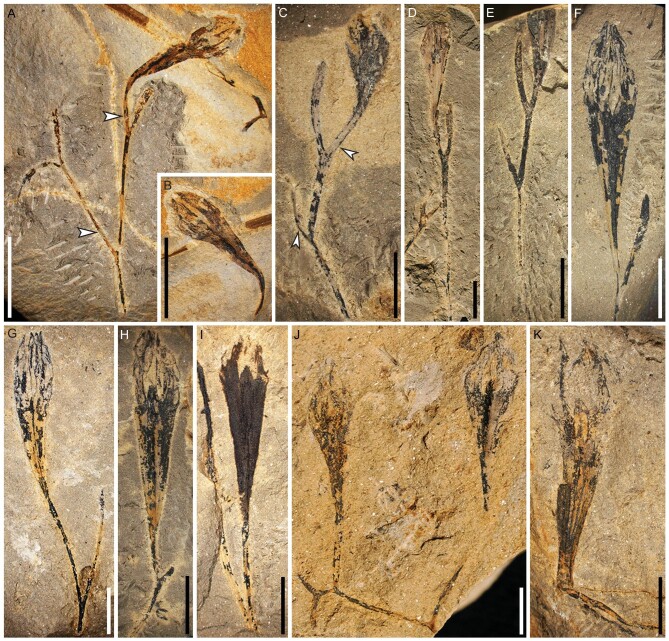
Fertile branches of *Guazia dongzhiensis*. (A) Twice dichotomizing branch with two axes (arrows) in different sedimentary planes and with a terminal ovule (PKUB14701a). (B) Counterpart of the terminal ovule in (A) (PKUB14701b). (C) Twice dichotomizing branch with two axes (arrows) in different sedimentary planes and a terminal ovule distally incomplete (PKUB14621). (D, E) Twice dichotomizing branch with one axis bearing a terminal ovule (PKUB14756, PKUB14768). (F–I) Once dichotomizing branch with one axis bearing a terminal ovule (PKUB14638, PKUB14699, PKUB14702, PKUB14601). (J) Thrice dichotomizing branch with two terminal ovules (PKUB14771). (K) Ovule terminating an ultimate axis (PKUB14705). Scale bars, 1 cm (A, B), 5 mm (C–K).

Holotype: PKUB14701a, b (part and counterpart housed in the Department of Geology, Peking University, Beijing), dichotomous axes with an acupulate ovule borne terminally (Fig. 1A and B).

Paratypes: PKUB14638, PKUB14699, PKUB14702, PKUB14601, PKUB14771, PKUB14705, terminal ovules on dichotomous axes (Fig. 1F–J) and on a short ultimate axis (Fig. 1K), respectively.

Locality and horizon: Xiangyu, Dongzhi, Anhui, China; Leigutai Member of the Wutong Formation, Upper Devonian.

Diagnosis: Dichotomous fertile branches bearing terminal ovules. Ovules elongate, obovoid, radially symmetrical and acupulate. Four broad wing-like lobes as integumentary outgrowths cruciately arranged in each ovule, distally tapered and proximally reduced. Individual integumentary lobes folding inwards along abaxial side. Free parts of integumentary lobes 30%–40% of ovule length. Nucellus except near apex adnate to integument. Nucellar apex dome-shaped.

### Description

**Figure 2.**
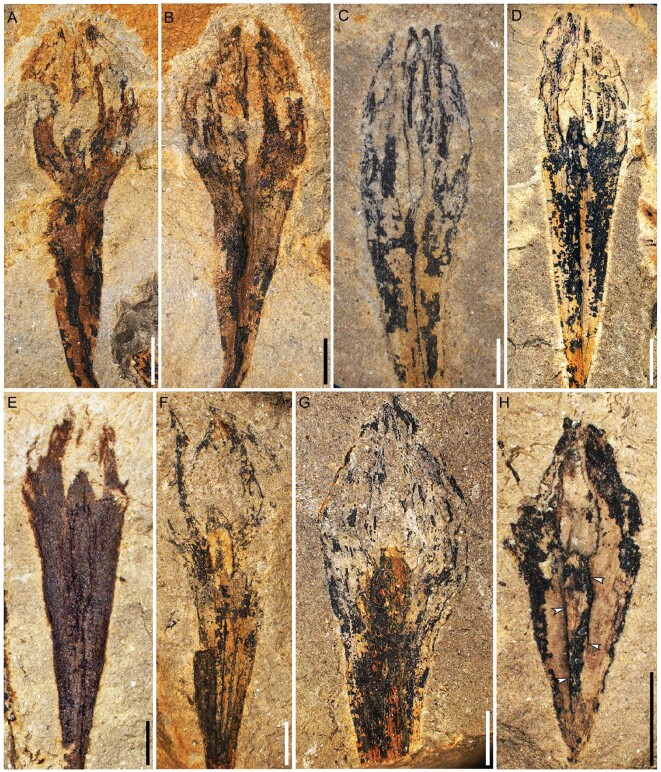
Ovules of *G. dongzhiensis*. (A–F) Enlargement of ovules in Fig. 1A, B, G–I and K, respectively. (B) Counterpart of (A). (G) Proximally incomplete ovule (PKUB14612). (H) Ovule showing attachment (arrows) of two integumentary lobes (PKUB14644). Scale bars, 2 mm.

Fertile axes are preserved with multiple orders of branching, and the isolated fossils examined show one to three orders of dichotomies. They are twice (Fig. 1A–E) or once (Fig. 1F–I), and rarely thrice (Fig. 1J) dichotomously branched at angles of 20–90°. The longest branching system excluding the ovules is 29.6 mm (Fig. 1A). The internodes are 2.0–18.8 mm long and the fertile axes are 0.2–1.0 mm wide. The branching is probably three-dimensional because two axes produced by successive dichotomies depart at different angles (Fig. 1A, arrows, and C, arrows), with one axis plunging into the rock matrix (Fig. 1A, upper arrow, and C, lower arrow). Some ultimate axes are terminated by a single ovule, whereas others lack ovules or are distally broken (Fig. 1). The boundary between the ultimate axis and ovule is difficult to distinguish, but it may be at the point where the basipetal reduction of width ceases (Fig. S2, arrows). The ultimate axes bearing ovules are 2.8–15.2 mm long and 0.3–0.8 mm wide. In most cases the ovules are detached, and either preserved with the distal part of the ultimate axis (Fig. 1K, Fig. S3A–E) or not (Fig. S3F–J). The ratio of length to maximum width of an ovule ranges from 2.6–(3.7)–4.6. The maximum width occurs at the mid-upper part of an ovule. Ovules are obovoid (Figs 1 and 2, Fig. S3), 7.8 mm (Fig. 2H) to 20.5 mm (Fig. 1H) long, and have a maximum width of 3.0–5.6 mm.

**Figure 3.**
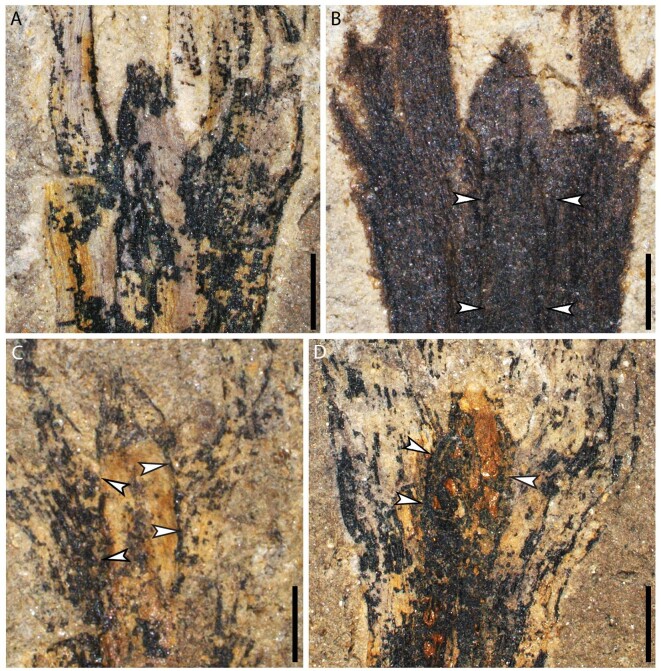
Ovules of *G. dongzhiensis*. (A–D) Enlargement of ovules in Fig. 2D–G, respectively, showing nucellar apex and attachment of integumentary lobes. (A) Ovule in Fig. 2D after dégagement. (B–D) Nucellus adnate to integument (arrows). Scale bars, 1 mm.

**Figure 4.**
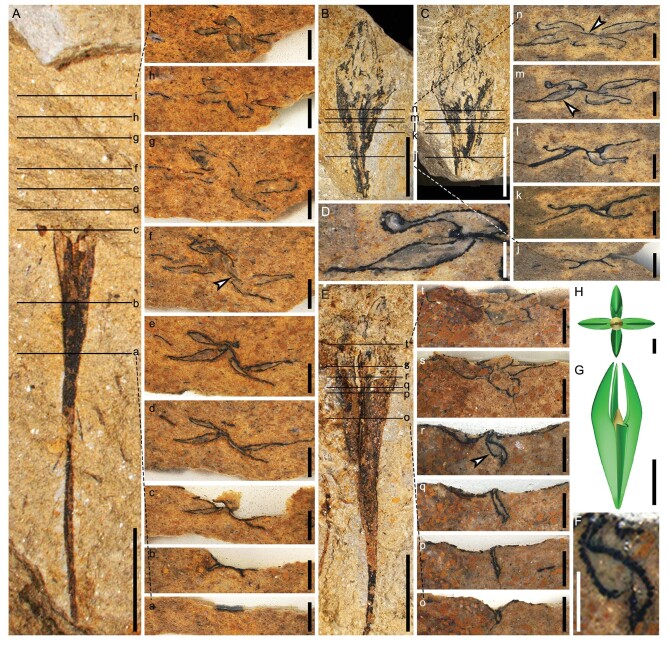
Transverse sections and reconstruction of ovules of *G. dongzhiensis*. (A) Ovule sectioned at nine levels (marked by lines a–i). Note the upper part of the ovule buried in rock matrix. (a–i) Nine transverse sections of ovule in (A) (at lines a–i, in ascending order), respectively. (B, C) Part and counterpart of an ovule, sectioned at five levels (marked by lines j–n). (j–n) Five transverse sections of ovule in (B, C) (at lines j–n, in ascending order), respectively. (f, n) Arrow indicating nucellar apex. (D) Enlargement of two arrowed integumentary lobes in (m). (E) Ovule sectioned at six levels (marked by lines o–t). (o–t) Six transverse sections of ovule in (E) (at lines o–t, in ascending order), respectively. (F) Enlargement of arrowed integumentary lobe in (r). (G) Lateral view of reconstructed ovule with one of four wing-like integumentary lobes partially removed. (H) Top view of ovule in G, showing nucellar apex and three of four integumentary lobes that curve centripetally at tips. Scale bars, 5 mm (A–C, E, G), 1 mm (a–t, H), 0.5 mm (D, F).

High magnification (Fig. S2), careful dégagement (Fig. 3, Figs S4–6) and serial transverse sections (Fig. 4A–F, a–t, Figs S7–11) of specimens do not show any cupules on terminally borne or detached ovules. Each ovule possesses a layer of integument with four prominent and radially extended lobes, which are broad in face view, distally tapered and proximally reduced to merge with the ultimate axis (Figs 2–4, Figs S2–11). The integumentary lobes are 1.2–2.4 mm at the maximum width and free for 2.4–7.5 mm (30%–40% of the ovule length), with the free parts of the integumentary lobes extending well above the nucellar apex and often curving centripetally. Usually, two integumentary lobes of a single ovule are evident (Figs S4A–D, 5B, C), while the other one (Figs S4d, 5b) or two lobes (Figs S4a–c, 5c) are more exposed through dégagement. This indicates that the integumentary lobes of an ovule are present on several bedding planes. The fusion of the integumentary lobes or outline of nucellus is either visible (Figs 2H, arrows, and 3, arrows), or suggested by dotted lines (Figs S4, 5A_,_ B, a–c). The integumentary lobes on several bedding planes may appear narrow or wide (Figs S4C, c, 5C, c), suggesting that they originally departed in different directions and extended cruciately in life (Fig. 4G and H).

In transverse sections of the ovules, two opposite integumentary lobes extend along the bedding plane and were originally perpendicular to the other two opposite lobes (Fig. 4b–n, Figs S8H–Z, a, b, 9D–W, 10a–g, 11A–J, a–f). During diagenesis, however, the other two lobes were usually compressed more or less along the bedding plane. Sections of an ovule show that one integumentary lobe is perpendicular to two opposite lobes (Fig. 4o–r, Fig. S10D–J) and then compressed (Fig. 4s and t, Fig. S10K–P). Multiple serial sections demonstrate that the four radially arranged integumentary lobes are narrow in the lower part of an ovule, becoming gradually wider towards the nucellar apex, narrowing again after overtopping the nucellus, and finally converging (Figs S7A–C, 8H–Z, a–d, 9D–W).

**Figure 5.**
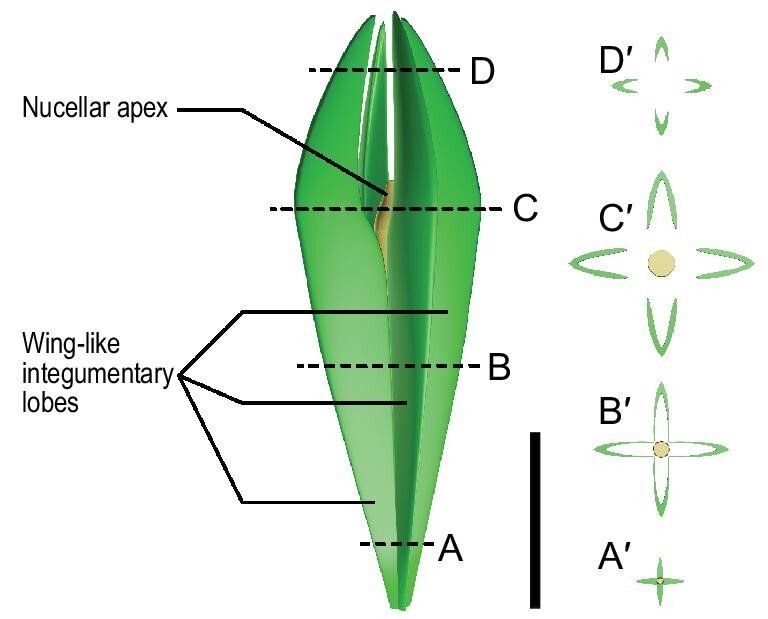
Reconstruction of the acupulate ovule of *G. dongzhiensis*. An ovule showing structures including nucellar apex and four wing-like lobes as integument outgrowths. Dotted lines A–D corresponding to the levels of four transverse sections A^′^– D^′^ of the ovule, respectively. Dotted circles in A^′^ and B^′^ indicating location of nucellus. Scale bar, 5 mm.

In transverse sections of the ovules, the individual integumentary lobes are flattened in the lower parts of an ovule (Figs 4b, j, 5A–A^′^, Figs S10B, C, a–g, 11a), become thick upwards (Figs 4c–e, k, l, o–r, 5B–B^′^, Fig. S10h), present a large V or U shape when free near the nucellar apex (Figs 4f, g, m, n, s, t, 5C–C^′^, Fig. S11A–E, b, c), form a small V or U shape over the nucellar apex (Figs 4h, i, 5D–D^′^, Figs S11F–I, d–f), and become flattened distally (Figs S8c, d, 11J). Therefore, the integumentary lobes are symmetrically folded along the abaxial side and toward the center. The surface of the integumentary lobes appears uneven with tiny indentations (Fig. 4D and F, Figs S8e, 9X, 11g), probably representing a parenchymatous outer layer of the integument (sarcotesta). These indentations and taphonomic compression sometimes result in the distortion and interruption of a single V- or U-shaped integumentary lobe in transverse section.

The nucellus may not be easily recognized in some ovules (Figs 1C–G, J, 2C, Figs S3, 7). However, well-preserved ovules and dégagement show that the nucellar apex is dome-shaped in lateral view, 1.0–1.7 mm long and 0.7–1.2 mm wide at the base (Figs 2D–H, 3, 4H, Figs S4–6). Transverse sections occasionally display the nucellar apex (Fig. 4f, arrow, and n, arrow). With the exception of the apex, the nucellus is closely fused to the integument (Figs 3, arrows, 4H, Figs S4, 5). The nucelli are at least 5.7 mm long (Fig. 2H), reaching ∼11.5 mm in length (Fig. 2D), with a maximum width of 0.8–1.4 mm, and distally surrounded by the free parts of the integumentary lobes. The ovule is reconstructed with the radially arranged integumentary lobes and nucellar apex (Fig. 5).

## DISCUSSION

The fertile fronds of the Late Devonian seed plants *Elkinsia* and *Dorinnotheca* branch dichotomously [16,17]. The ultimate axes with terminal cupulate ovules of *Elkinsia* are cruciately arranged in three dimensions, while those of *Dorinnotheca* are pinnately arranged and the ovules pendulous. The fertile branching of *Guazia* is, where known, also dichotomous. The ultimate axes are terminated by single ovules and there appears limited evidence that these axes occur in different planes.

Of Devonian ovules, *Guazia* most resembles *Pseudosporogonites* [18] in its radial symmetry and four broad integumentary lobes. However, the ovule of *Pseudosporogonites* is surrounded at the base by a collar-shaped cupule; the three or four integumentary lobes are only fused for 1/3 length of the integument and are tangentially arranged around a nucellus; the tips of the integumentary lobes and nucellus are located at approximately the same level. *Guazia* lacks a cupule, but possesses an integument that surrounds the nucellus (except for its apex) and produces four radially arranged outgrowths. These outgrowths are interpreted as wing-like integumentary lobes. It is sometimes difficult to distinguish the nucellus from the closely adpressed integument.

All Devonian ovules so far described for which attachment is known are surrounded by a cupule [4,18] that was presumably involved in both protection and pollination [2,5,19]. The wing-like ovules of *Warsteinia* are detached and the cupule unknown [7], but they are assumed to have been cupulate as in other Devonian ovules [18]. The ovules of *Guazia* are attached to branches and clearly lack a cupule, providing the first convincing evidence for acupulate ovules in the earliest seed plants. The telome theory proposes that the cupule evolved from the coalescence of the surrounding vegetative dichotomous branches [2,20]. In this context, the absence of cupules in *Guazia* might have resulted from a reduction of these branches.

Cupules disappeared in the Carboniferous (Late Mississippian)–Permian medullosan spermatophytes and later in several other derived lineages; instead, the importance and complexity of the integument increased [2,4]. Like *Guazia*, some medullosan ovules are borne terminally on dichotomous branches and possess four integumentary wings [21]. Among Devonian ovules, *Pseudosporogonites* and *Latisemenia* have a very short cupule only surrounding the ovule base [18,22]. The integumentary lobes of these two genera, especially *Latisemenia*, are so broad and largely fused as to effectively protect the nucellus. The nucellus (except at the tip) of *Guazia* is adnate or close to the integument and the free portions of the integumentary lobes extend a long distance above the nucellar apex, fold inwards and often curve centripetally. In the absence of a cupule, such an integument highlights its role in protection. In contrast, the free portions of the integumentary lobes of *Warsteinia* are short, flat, straight and open. The integumentary lobes of early ovules suggest pollen capture by wind, i.e. anemophily as a pollination syndrome [2]. Thus, the integument of *Guazia* appears to have the same structural function of protection and pollination as the cupule. *Guazia* may also perform the function of potential wind dispersal by wing-like integumentary lobes, despite losing the protective and pollinating functions of the cupule (see below).

Winged diaspores (e.g. seeds or fruits) of fossil and living seed plants vary greatly in size but can promote (horizontal) wind movement during their abscission from the source plant, especially if given enough height [2,23–26]. In addition, having landed, a winged diaspore may be blown along the ground, a movement termed secondary wind dispersal [27]. Late Devonian *Warsteinia* with four wing-like integumentary lobes suggests its possible adaptation for wind dispersal; the lobes increase floatation and saltation/adherence before and after landing, respectively [7,8]. *Guazia* is the second Devonian ovule with such radially arranged lobes. The lobes appear thin as in *Warsteinia*, however, fold lengthwise and usually arch centripetally above the nucellar apex. This ovulate morphology of *Guazia* may not have been much effective in primary dispersal (airborne movement), but could potentially facilitate the secondary dispersal by wind. Most specimens of *Guazia* (ca. 90% collections) were found as detached ovules with ultimate axis tips sometimes preserved. They provide clear evidence that the ovules were shed, and the narrow connection of the ultimate axis to the ovule suggests the likely point of disarticulation.

## MATERIALS AND METHODS

All specimens in this study were collected in 2006–2008, and are deposited in the Department of Geology, Peking University, Beijing, China. Steel needles were used to expose some fertile axes and ovules. Serial dégagement was employed to reveal the morphology and structure of the ovules. Ovules were embedded in resin, sectioned and ground to display the integumentary lobes. Maceration of the specimens produced only fragments of organic matter, so we have failed to see some details of the ovules (e.g. megaspore). All photographs were taken with a digital camera and Leica microscope. Using Autodesk 3ds Max 2017 software, we generated the reconstruction of the ovule (Figs 4G and H, 5).

## Supplementary Material

nwab196_Supplemental_File
