# Supplementary material for: Guazia, the earliest ovule without cupule but with unique integumentary lobes
Source: Natl Sci Rev. 2021 Oct 30;9(4):nwab196. doi: 10.1093/nsr/nwab196 (PMC8982201; doi:10.1093/nsr/nwab196)
Supplement: nwab196_Supplemental_File [file nwab196_supplemental_file.pdf]

Supplementary Data for

Guazia, the earliest ovule without cupule but with unique integumentary lobes

De-Ming Wang[1]†*, Le Liu[2]†, Yi Zhou[1]†, Min Qin[3], Mei-Cen Meng[4], Yun Guo[5]*, Jin-Zhuang Xue[1]

[1]Key Laboratory of Orogenic Belts and Crustal Evolution, Department of Geology, Peking University, Beijing 100871, China.

[2]School of Geoscience and Surveying Engineering, China University of Mining and Technology (Beijing), Beijing 100083, China.

[3]Institute of Geology and Paleontology, Linyi University, Linyi 276000, China.

[4]Science Press, China Science Publishing and Media Ltd., Beijing 100717, China.

[5]Institute of Deep Time Terrestrial Ecology, Institute of Palaeontology, Yunnan Key Laboratory of Earth System Science, Yunnan University, Kunming 650500, China.

†These authors contributed equally to this work.

*Corresponding authors. Email: dmwang@pku.edu.cn (D.-M.W.); guoyun@ynu.edu.cn (Y.G.)

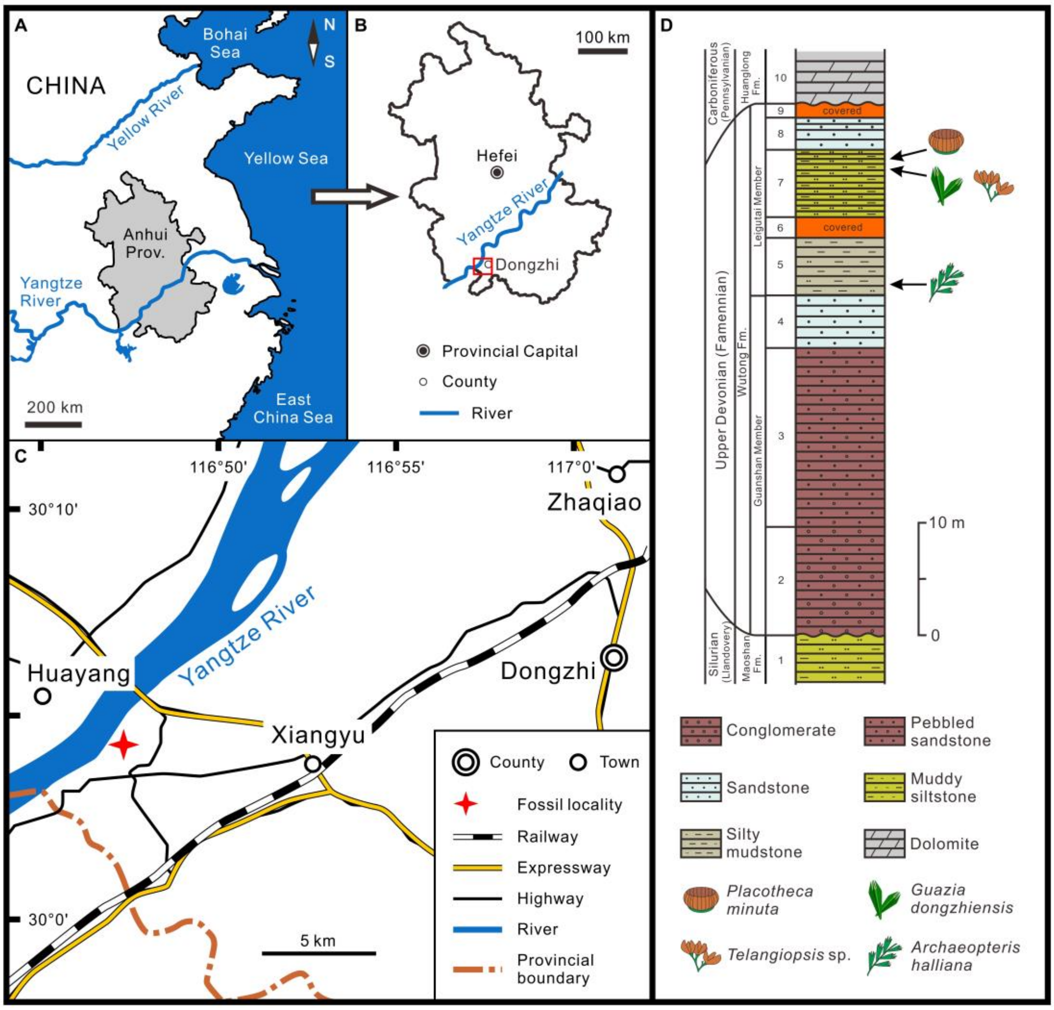

Fig. S1. Map showing the locality and stratigraphy of the fossil ovule *Guazia dongzhiensis*. (A-C) Locality of ovules at Xiangkou Section, Xiangyu, Dongzhi, Anhui, China. (C) Enlargement of red rectangular area in B. (D) Stratigraphic column of the Wutong Formation at Xiangkou Section, showing the lithology and occurrence of *G. dongzhiensis*, pollen organs *Placotheca* and *Telangiopsis*, and progymnosperm *Archaeopteris*.

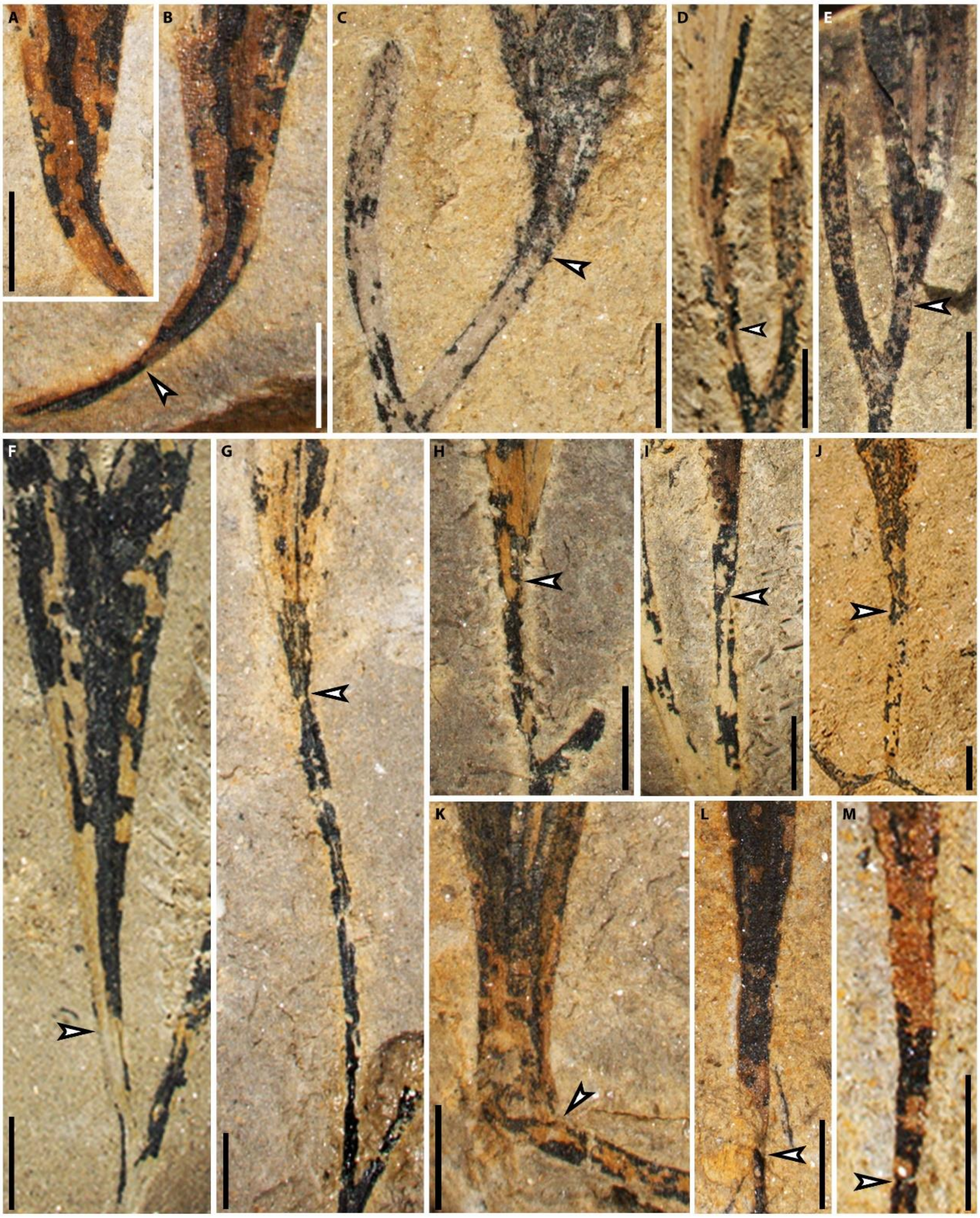

Fig. S2. Base of ovules of *G. dongzhiensis*. (A-M) Enlargement of ovules in Figs. 1A-K, 4A, E, respectively, showing ovule base connected to ultimate axis and absence of cupule. Arrows indicating suggested boundary between ovule and axis. Scale bars, 2 mm.

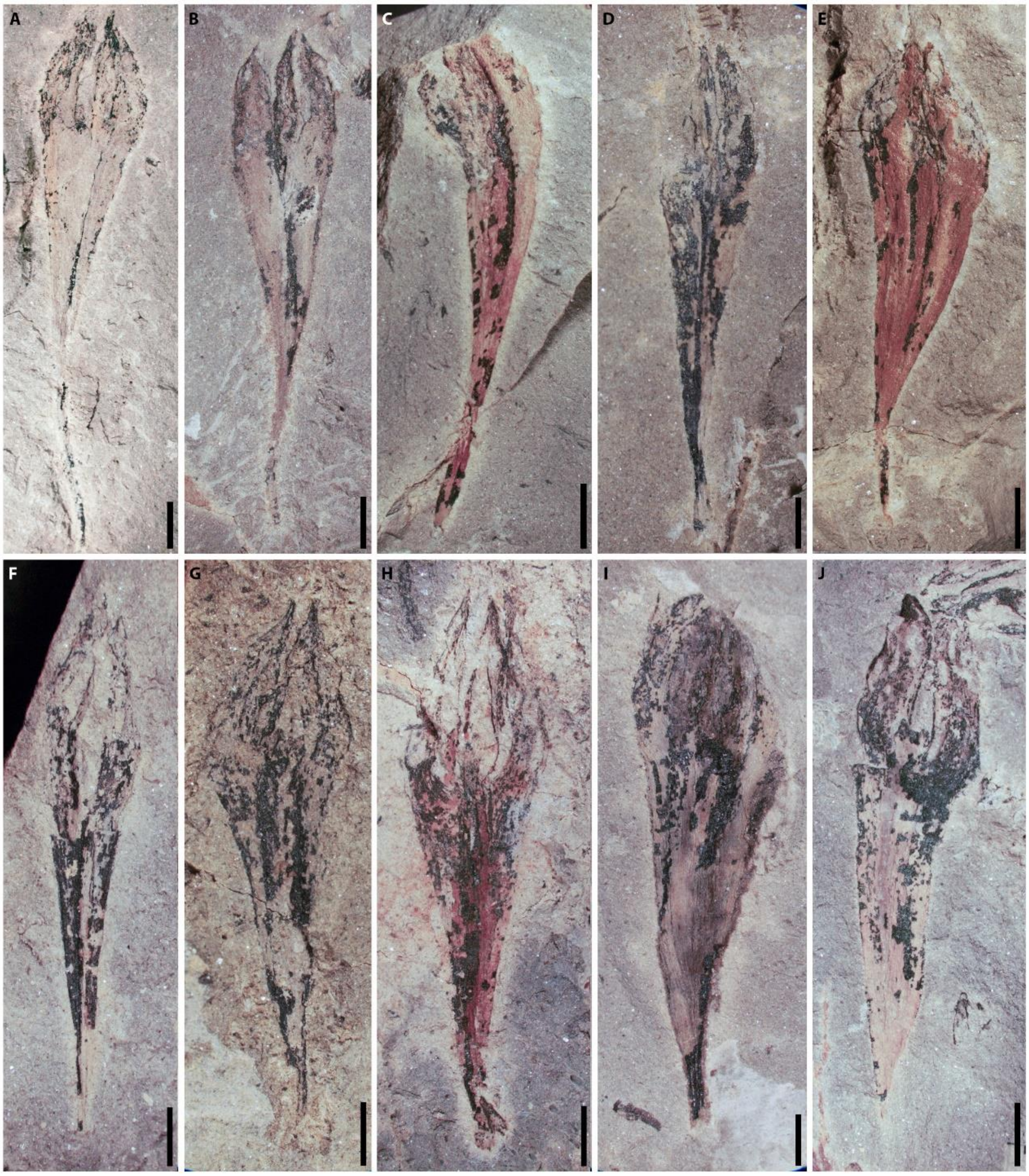

Fig. S3. Detached ovules of *G. dongzhiensis*. (A-E) Ovules connected with distal part of ultimate axis (PKUB14614, PKUB14604, PKUB14605b, PKUB14638, PKUB14614). (F-J) Ovules lacking distal part of ultimate axis (PKUB14690, PKUB14669, PKUB14628, PKUB14671, PKUB14694). Scale bars, 2 mm.

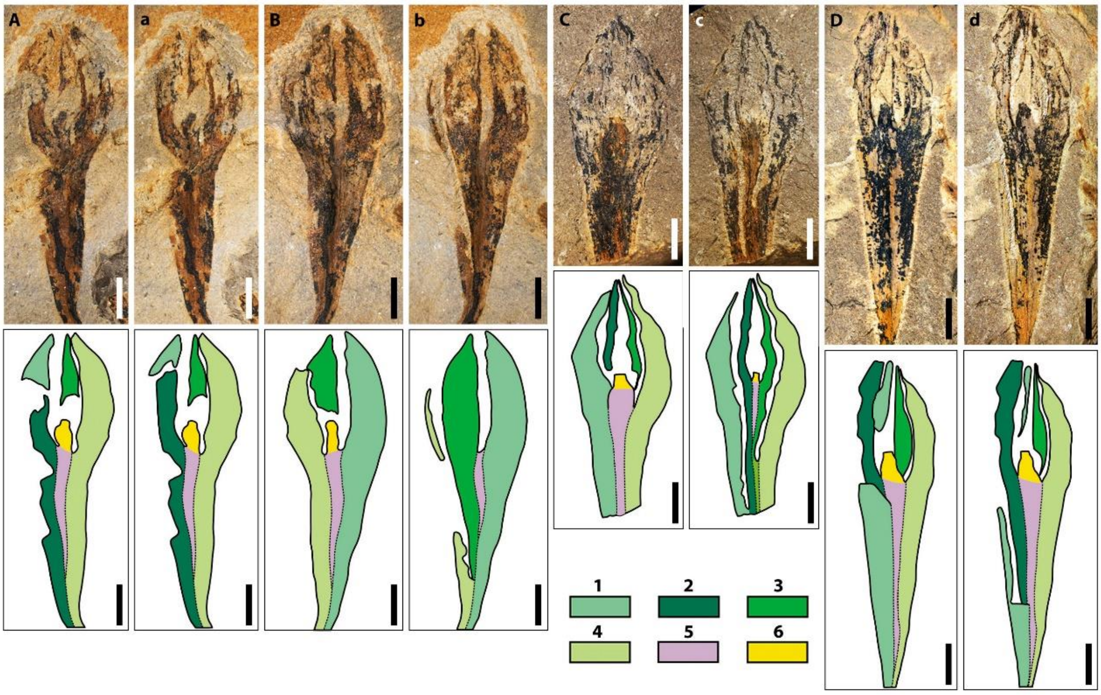

Fig. S4. Dégagement of ovules of *G. dongzhiensis*. (A-D) Ovules in Fig. 2A, B, G, D and their line-drawings, respectively. (a-d) Dégagement of ovules in (A-D) and their line-drawings, respectively. Colours 1-4, four integumentary lobes of an ovule; colour 5, integument adnate to nucellus; colour 6, apex of nucellus; dotted line, indicating fusion of integumentary lobes or outline of nucellus. Scale bars, 2 mm.

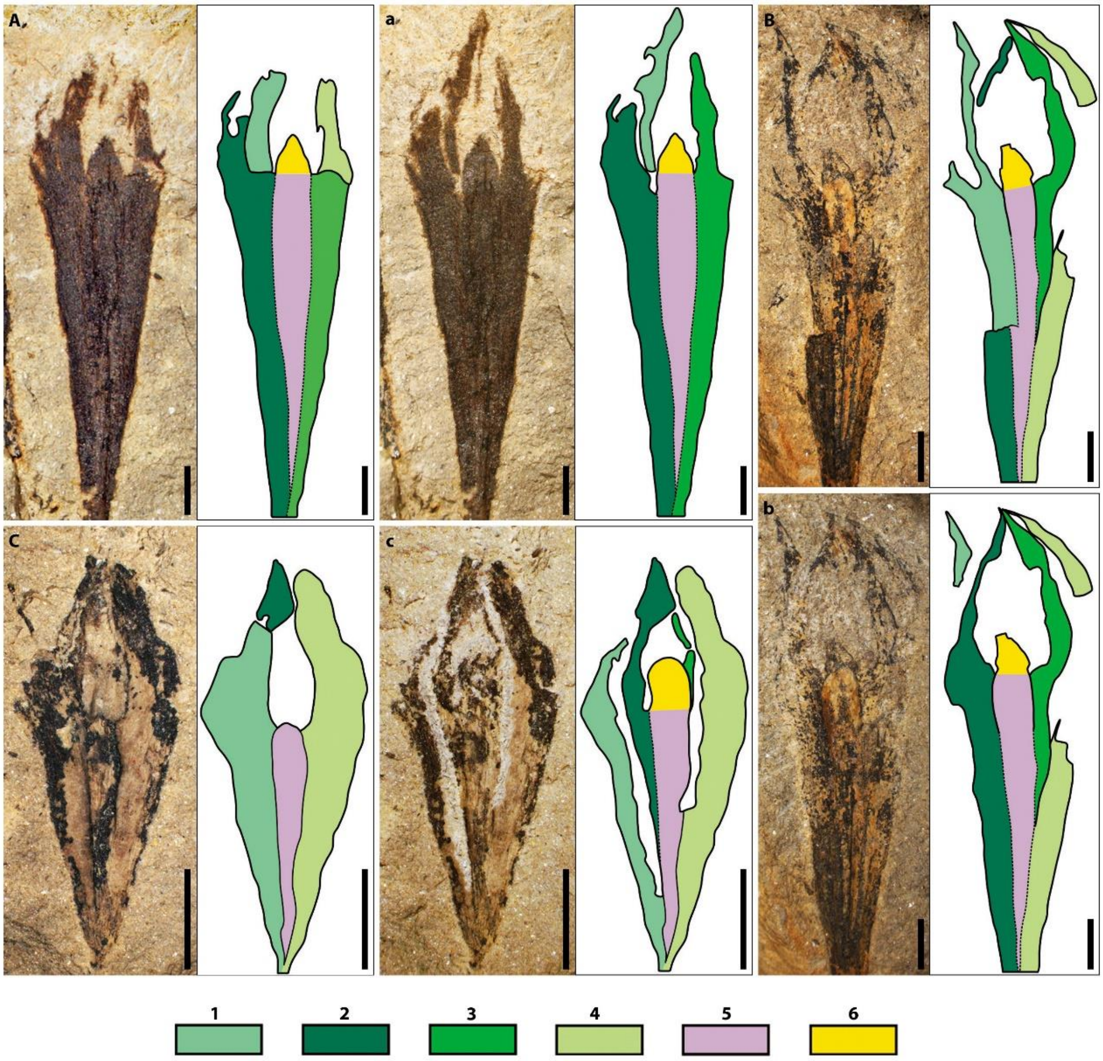

Fig. S5. Dégagement of ovules of *G. dongzhiensis*. (A-C) Ovules in Fig. 2E, F, H and their line-drawings, respectively. (a-c) Dégagement of ovules in (A-C) and their line-drawings, respectively. Colours 1-4, four integumentary lobes of an ovule; colour 5, integument adnate to nucellus; colour 6, apex of nucellus; dotted line, indicating fusion of integumentary lobes or outline of nucellus. Scale bars, 2 mm.

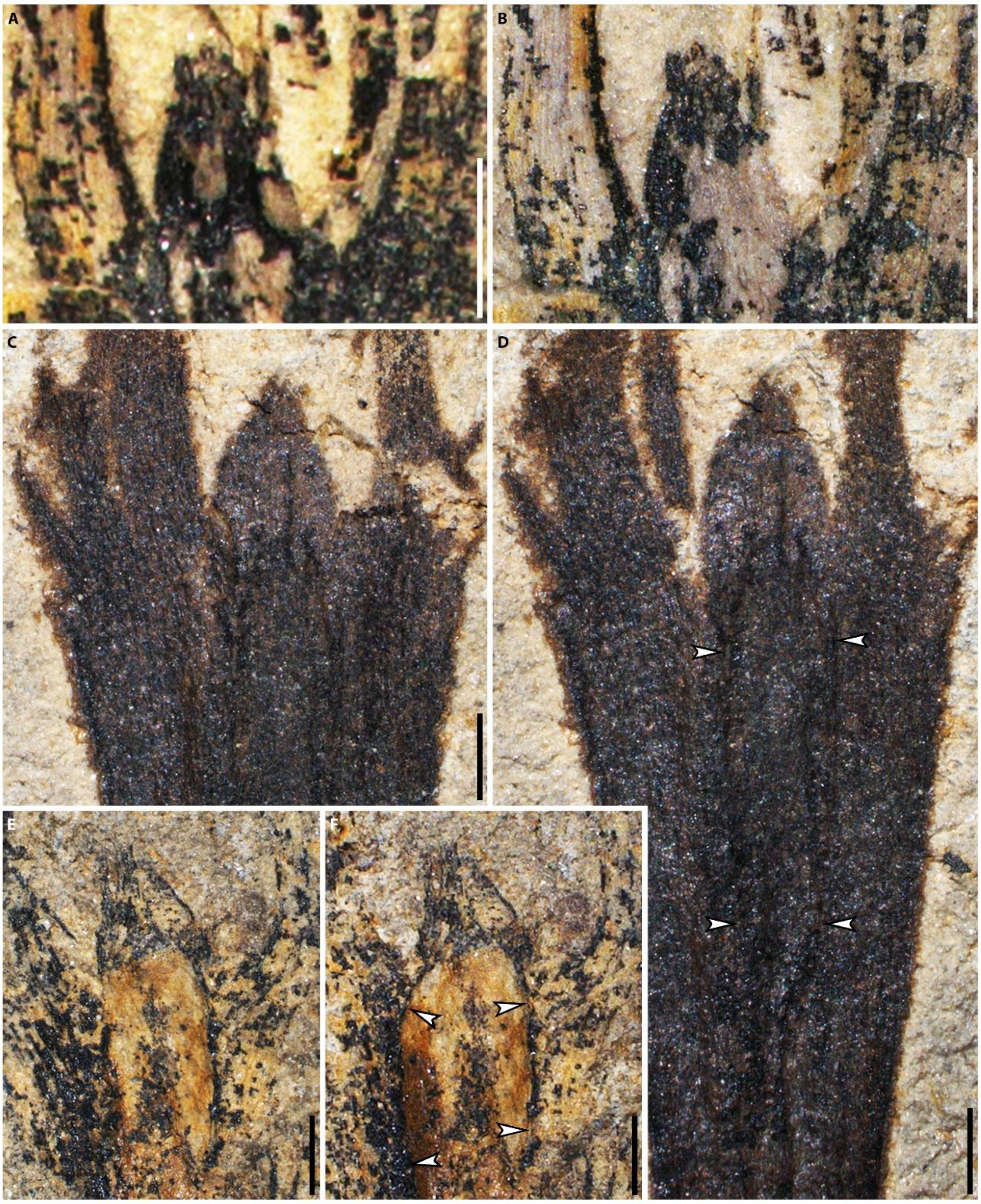

Fig. S6. Ovules of *G. dongzhiensis*. (A, B) Enlargement of nucellus in Fig. S4D, d, respectively. (C-F) Enlargement of ovules in Fig. S5A, a, B, b, respectively, showing nucellus adnate to integument (arrows). Scale bars, 1 mm.

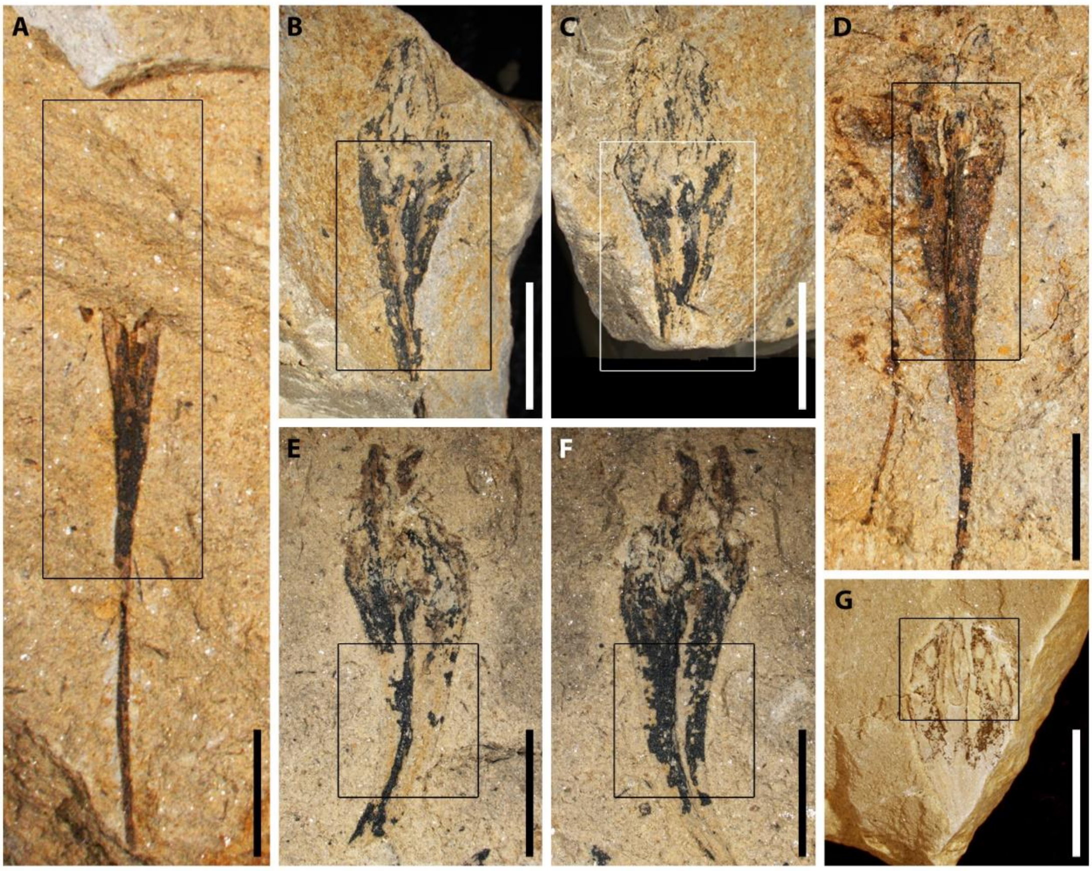

Fig. S7. Ovules of *G. dongzhiensis*. (A-G) Ovules used for transverse sections. (A) Ovule as in Fig. 4A. (B, C) Part and counterpart of an ovule as in Fig. 4B, C. (D) Ovule as in Fig. 4E. (E, F) Part and counterpart of an ovule. Rectangles, regions where ovules were transversely sectioned. Scale bars, 5 mm.

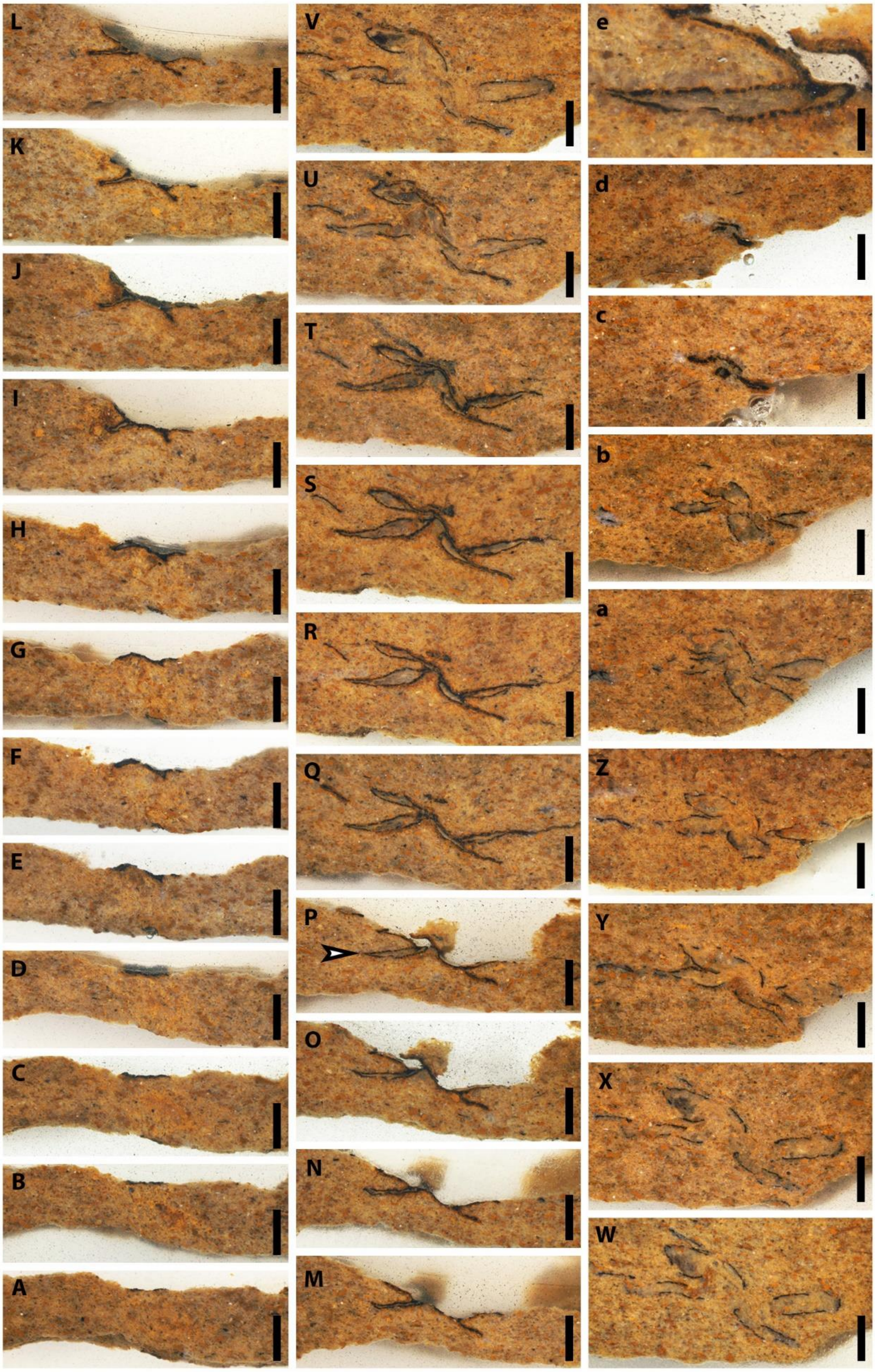

Fig. S8. Transverse sections of ovule of *G. dongzhiensis*. (A-Z, a-d) Serial sections of an ovule in Fig. S7A (rectangular area, in ascending order). (e) Enlargement of arrowed integumentary lobe in (P). Scale bars, 1 mm (A-Z, a-d), 0.2 mm (e).

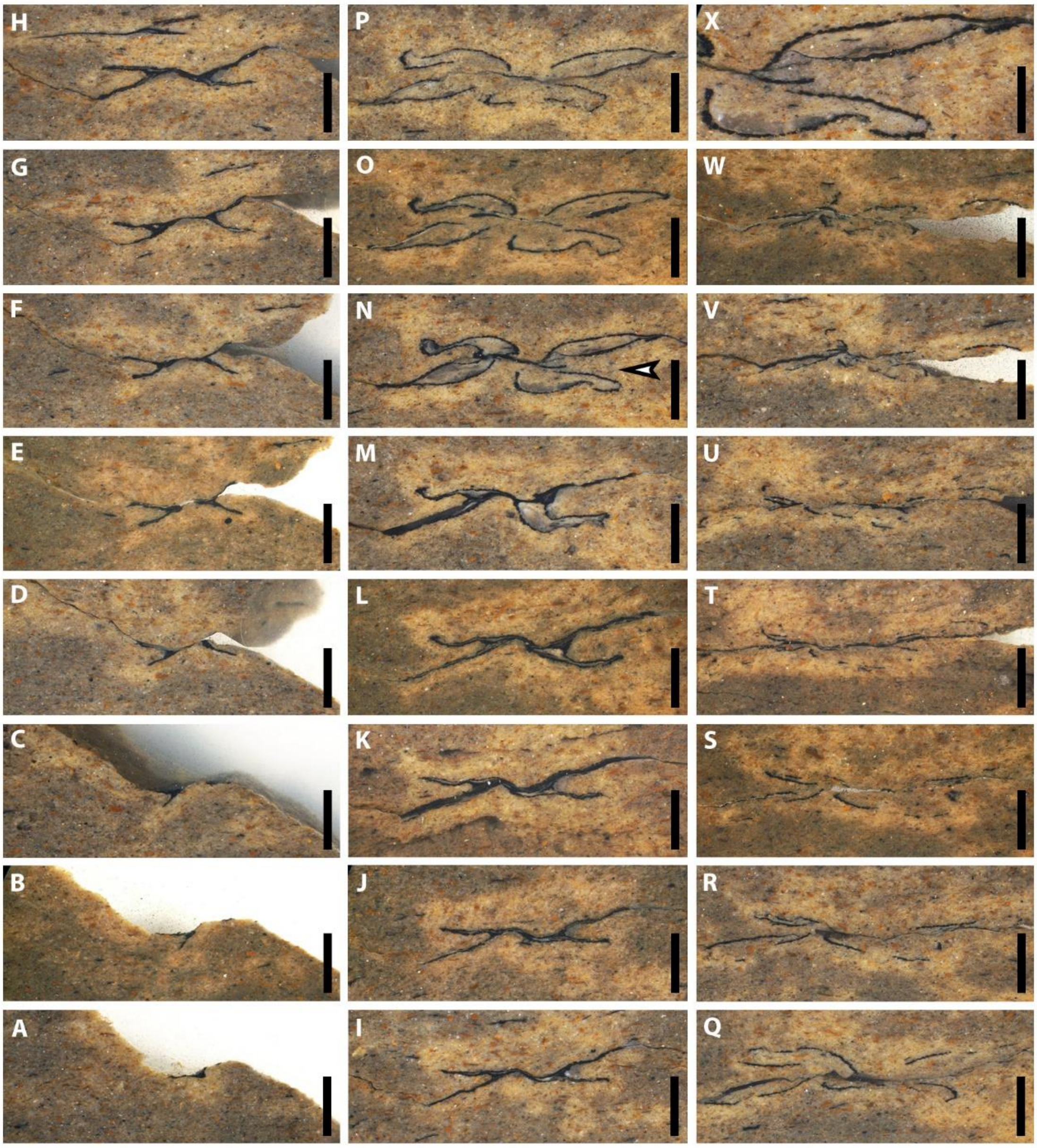

Fig. S9. Transverse sections of ovule of *G. dongzhiensis*. (A-W) Serial sections of an ovule in Fig. S7B, C (rectangular area, in ascending order). (X) Enlargement of two arrowed integumentary lobes in (N). Scale bars, 1 mm (A-W), 0.5 mm (X).

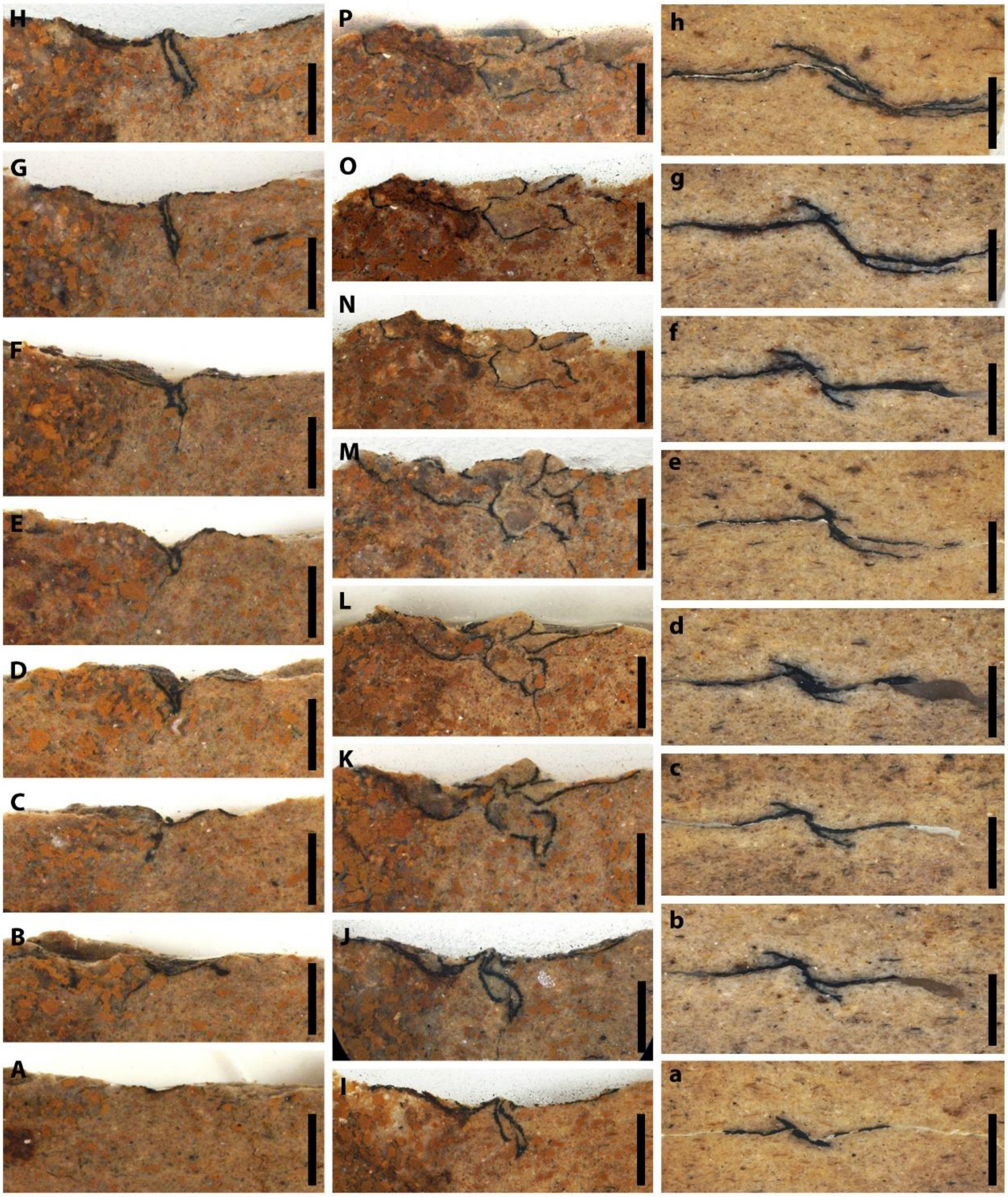

Fig. S10. Transverse sections of two ovules of *G. dongzhiensis*. (A-P) Serial sections of an ovule in Fig. S7D (rectangular area, in ascending order). (a-h) Serial sections of an ovule in Fig. S7E, F (rectangular area, in ascending order). Scale bars, 1 mm.

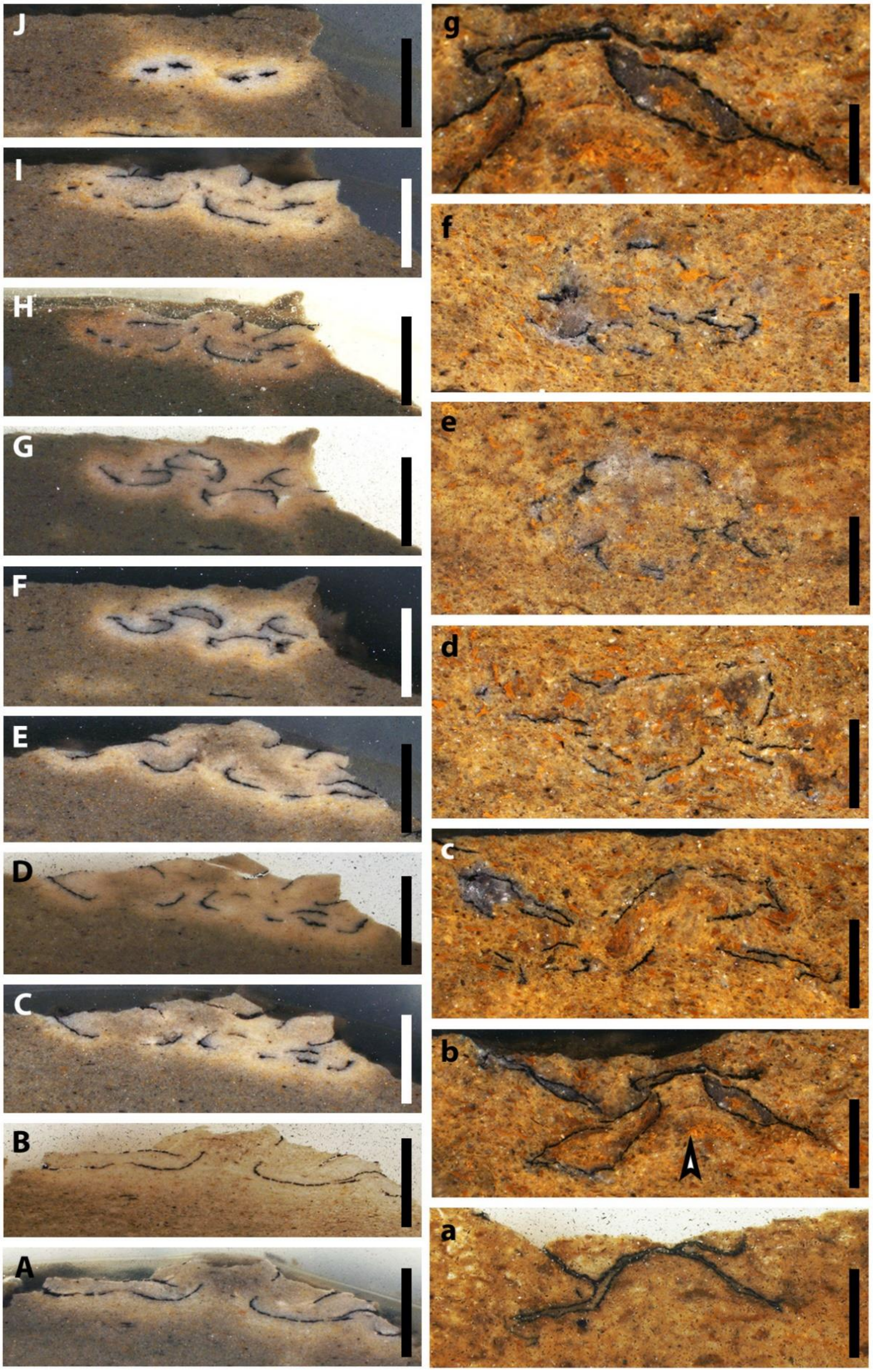

Fig. S11. Transverse sections of two ovules of *G. dongzhiensis*. (A-J) Serial sections of an ovule in Fig. S7G (rectangular area, in ascending order). (a-f) Serial sections of an ovule from base to tip. (g) Enlargement of arrowed integumentary lobes in (b). Scale bars, 1 mm (A-J, a-f), 0.5 mm (g).
